# Examining the role of affective states in relation to exercise intentions and participation in extra-curricular exercise classes at university: A repeated measurement observational study

**DOI:** 10.3389/fpsyg.2022.815466

**Published:** 2022-08-22

**Authors:** Emily Finne, Carina Nigg, Susanne Weyland, Odile Sauzet, Benjamin Wienke, Darko Jekauc

**Affiliations:** ^1^School of Public Health, Bielefeld University, Bielefeld, Germany; ^2^Institute of Sports and Sports Science, Karlsruhe Institute of Technology, Karlsruhe, Germany; ^3^Center for Statistics (ZeSt), Bielefeld University, Bielefeld, Germany; ^4^Institute for Sport Sciences, Humboldt University of Berlin, Berlin, Germany

**Keywords:** exercise, core affect, affective states, affective valence, intention, behavior

## Abstract

**Background:**

Previous research has shown evidence on the role of affective states for physical activity behavior. However, there is a lack of research investigating the interplay between affective states, intentions, and exercise behavior, especially with respect to maintaining regular exercise over time. The study aimed to investigate whether post-exercise affective states and changes in affect during exercise (i) are related to exercise intentions; (ii) moderate the relationship between intention and subsequent exercise behavior, and (iii) directly predict future exercise.

**Methods:**

Participants from weekly voluntary sports and gym classes at two universities were recruited. For 13 weeks, 268 individuals’ (M*_age_* = 24.5 years, SD = 5.6, 90% students, 67.4% female) class attendance was documented on a weekly basis. Before and immediately after training, participants self-reported affective states, including affective valence (Feeling Scale) and perceived arousal (Felt Arousal Scale). Participants also reported their intention to re-attend the class the following week. Mixed-effect linear models and Cox proportional hazard models were used to examine the relationships between affective states, change in affective states, re-attendance intentions, and class re-attention.

**Results:**

Affective valence at the end of training was significantly positively associated with the intention to re-attend the class on the within-person level (β = 0.880, *p* < 0.001) as well as the between-person level (β = 0.831, *p* < 0.001), while higher increases of valence during class were related to smaller intention. For class re-attendance, significant effects of affective states were only found on the within-person level. A one-point increase on the valence scale increased the hazard ratio to re-attend by 8.4% (*p* < 0.05), but this effect was no longer meaningful after adjusting for intention. No moderation of the relationship between intention and subsequent class re-attendance was found.

**Conclusion:**

The results suggest that positive affective state immediately after exercise does not facilitate translation of intentions into subsequent exercise behavior (i.e., do not close the intention-behavior gap). Rather, affective valence was found to be an important predictor of exercise intentions but seemed indirectly related to behavior *via* intentions. Practitioners should plan exercise programs that allow for positive affective states especially at the end of a training.

## Introduction

Numerous studies have shown evidence for the positive associations between physical activity (PA) and health, such as a reduced risk for breast and colorectal cancer, coronary heart disease, cardiovascular disease, and overall mortality (Lee et al., 2012; Reiner et al., 2013; Warburton and Bredin, 2017). Sustainable health improvements can only be achieved by regularly accumulating the recommended amount of PA (Haskell et al., 2007; Powell et al., 2011). However, only 22.6% of adults in Germany meet the recommendations for aerobic and muscle strengthening PA (Finger et al., 2017; World Health Organization, 2020). Moreover, maintaining regular participation in PA seems to be difficult for many people, as has been shown through high rates of relapsing into lower activity levels within months or even weeks after having started to exercise (Annesi, 2003; Finne et al., 2019).

If the objective of engaging in structured PA is the improvement or maintenance of at least one component of physical fitness, it is defined as exercise (Caspersen et al., 1985). In past health behavior and exercise research, social-cognitive approaches were the predominant explanations of behavior and behavior change (Jekauc et al., 2015; Rhodes et al., 2019). A central social-cognitive construct is intention, which plays a crucial role, for example, in the widely used Theory of Planned Behavior (TPB; Ajzen, 1991). Intention is described as representing a person’s motivation and behavioral orientation toward a behavior (Hagger et al., 2002). Intention is a necessary prerequisite of PA adoption, as almost nobody reports PA implementation without intention (Rhodes and Bruijn, 2013), and intention has been shown to be an important predictor of PA behavior (McEachan et al., 2011; Rhodes and Bruijn, 2013).

However, intentions leave a large part of behavioral variance unexplained. For example, a meta-analysis showed that only 42% of people translate PA intentions into PA behavior, 36% do not engage in PA although they intend to do so, while the rest expressed no intention (Rhodes and Bruijn, 2013). This discrepancy between behavioral intention and actual behavioral implementation is often described as intention-behavior gap. Whether intentions are implemented or not may also depend on additional psychological factors moderating the intention-behavior relationship (Sniehotta et al., 2005). In that sense, social-cognitive approaches have been increasingly criticized for focusing on rational decision making and disregarding non-reflective processes of motivation and behavior regulation, such as affective processes (Sniehotta et al., 2014; Hagger, 2016; Rebar et al., 2016).

Affect plays a central role in several current theoretical models of physical activity that take a dual process approach (Brand and Ekkekakis, 2018; Stevens et al., 2020; Strobach et al., 2020). These state that PA behavior is regulated by a reflective pathway mediated by higher cognitive processes as well as by an implicit and automatic pathway in which processes are mainly executed unconsciously.

According to the feedback theory of Baumeister et al. (2007), automatic affect can be understood as a simple and quick valuation of the situation, that something is pleasant or unpleasant. This is expressed in a feeling that is not reflective and suggests to us that the situation is good or bad, liking or disliking, to be approached or to be avoided. Although affect is seen as an automatic response, it contains a cognitive component, and the resulting feeling can be conscious. In this conception, “automatic affective responses can preserve the lessons and information from previous emotional experiences” (Baumeister et al., 2007, p. 172). This previous affective experience contributes to the anticipation of an affective state of a future event and influences the decision about future behavior (see in the context of physical activity, Feil et al., 2022). As similar concept, in the circumplex model of affect, the mental structure of core affect can be reflected by the two dimensions valence (pleasure vs. displeasure) and activation (high vs. low arousal; Russell, 1980, 2003). With its evaluative and reinforcing role, valence is considered the relevant affect dimension for influencing future behavior (Baumeister et al., 2007; Stevens et al., 2020). Valence is seen as the most basic building block of emotional life and might be a strong catalyzer for motivational processes (Barrett, 2006). The dimension of affective arousal on the other hand is seen as orthogonal dimension without a hedonic value. Therefore, arousal is expected to show minor importance as input for motivational effects compared to valence. There are indications, however, that arousal impacts attention and memory processes (Storbeck and Clore, 2008).

Affective states during the execution of a behavior or immediately after its completion are termed “affective response” within the Affect and Health Behavior framework (Williams and Evans, 2014; Stevens et al., 2020). According to this framework, affective responses are cognitively processed both automatically (non-reflective) and reflectively, resulting in affectively charged forms of motivation (non-reflective) on the one hand and behavioral intentions and goals (reflective) on the other hand. Thus, affect may directly influence subsequent behavior *via* the implicit pathway, or indirectly by informing reflective processes, leading to intentions in terms of future behavior.

The role of affective states within the dual process approach is also addressed by the Physical Activity Adoption and Maintenance (PAAM) model (Strobach et al., 2020). The authors assume an additional moderating effect of affective states on the relationship between intention and physical activity. It is hypothesized that intentions are more easily translated into action when accompanied by positive affective states (Strobach et al., 2020). Conversely, it is difficult to put an intention into action when affective resistance is high. The permanent overcoming of negative feelings requires a lot of energy to regulate affective states, so that the cognitive capacities of a person can deplete. This effect, also called ego depletion, increases the likelihood that intention will not be translated into action (Englert, 2017). Hence, as a third mechanism, affect may moderate the intention-behavior relationship and could in this way help to close the intention-behavior-gap.

According to the Law of Effect (Thorndike, 1911), positively experienced behavior has a higher probability of being repeated than behavior not associated with positive affective states. A number of empirical studies seem to support this hypothesis and substantiate the role of affect-related variables as determinants of physical activity behavior (for overviews see for example Rhodes et al., 2009; Rhodes and Kates, 2015; Stevens et al., 2020). However, not all studies consider the affective state (“core affect” or “affective response”) itself, and different affect-related variables are sometimes summarized under the term “affect.”

Stevens et al. (2020) review the literature according to the distinction of four affect-related concepts based on the Affect and Health Behavior Framework (Williams and Evans, 2014). Besides affective response to health behavior and incidental affect (not behavior-related), these rather reflect cognitive processing of the experienced affective states, resulting in reflective constructs like affective attitudes, enjoyment, expectations of future affective responses to behavior or affectively charged forms of motivation. Affective state itself can only be measured within a situation while experiencing it (Williams and Evans, 2014; Stevens et al., 2020).

Rhodes and Kates (2015) in their systematic review included 24 studies and found that positive affective responses during moderate exercise but not post-exercise affect were related to future PA, while relationships of affective responses with subsequent intentions were very small, albeit only few studies examined responses during exercise in this context. Furthermore, the majority of studies focused on cross-sectional associations. Very few studies so far have experimentally manipulated affect-related variables (see for example Jekauc, 2015 who measured enjoyment pre- and post-an intervention).

Several individual studies point to an influence on intentions. For example, Kwan and Bryan (2010a) found a small relationship between positive affective states during and post-exercise with intentions to exercise, measured 3 months later. In another study, Raedeke et al. (2007) found that post-exercise affective states, aside from distinct categorical feeling state responses to acute exercise, were significantly and substantially related to subsequent intentions for continued exercise behavior. A review (Rhodes and Kates, 2015), however, concluded that there might only be a negligible association between post- or during-exercise affect and exercise intention. Nevertheless, several of the included studies were small and lacked sufficient capability to detect smaller effects. Thus, there is some evidence for an effect pathway from affect to intentions, but the results are not conclusive (Rhodes et al., 2022). Moreover, most studies so far focused on between-subject effects and did not consider fluctuations of affect over time within subjects. Consequently, it should be noted that there is a dearth of studies that map the dynamics of affective states over weeks and analyze the relationship with intentions over time. Especially because affective states are highly volatile, it can be assumed that within-subject fluctuations are particularly important for the prediction of intentions and future behavior.

One purpose of our study was to examine the link between affective states and the intention to re-attend an exercise class at the next opportunity, with focus on the valence dimension of affect and the variation of within and between subjects. Another aim of this study was to examine the potential influence of affective states on the translation of intentions into behavior, that is, the potential moderating effect of affective states (Strobach et al., 2020).

**Figure 1:**
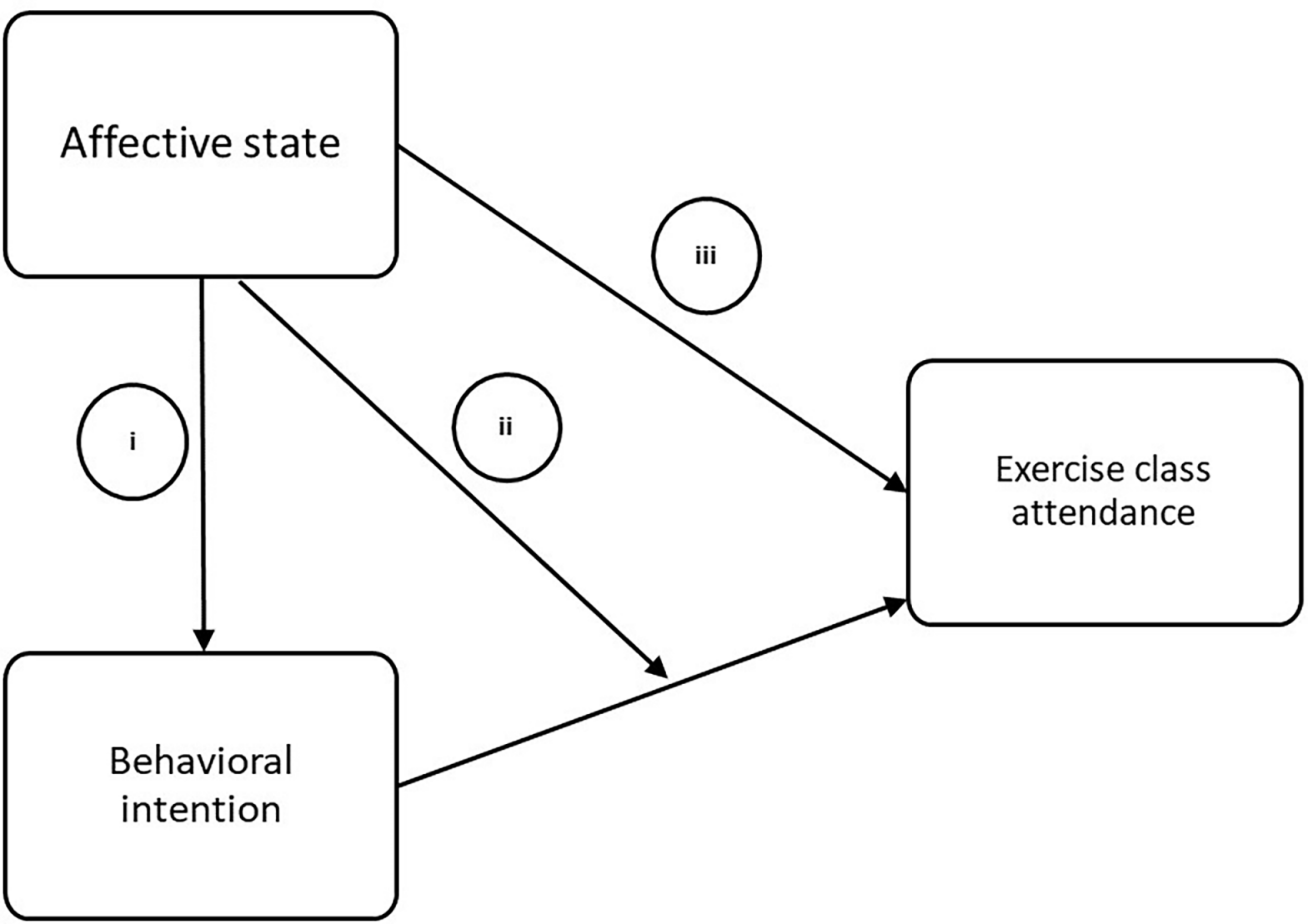
Research hypotheses tested in this study.

Derived from the outlined research findings and proposed theories, three different hypotheses about the interplay between affective states (valence) and behavioral intentions as determinants of exercise behavior, i.e., class re-attendance, have been proposed and tested (see Figure 1).

- (Positive) Affective states contribute to the formation of intentions.
- Affective states moderate the intention – exercise class re-attendance relationship.
- Affective states directly influence exercise class re-attendance.

## Materials and methods

### Participants

We recruited participants from 10 different sports and gym classes at two German universities during the winter semester 2015/2016. To be considered for this study, participants had to have baseline data and sufficient data from at least one of the weekly short questionnaires, resulting in 268 participants (*N* = 141 and *N* = 127 for the two universities, respectively). The sports and gym courses are offered for low fees by the department of collegiate sports to all students and employees of the universities. About 91% of the participants were students (*N* = 241), and 67.9% were females (*N* = 182). The mean age was 24.50 years (SD = 5.60, range = 16–57).

Classes are offered each semester, starting and ending identically to the lecture period. Participants must register for specific courses on time, and available spots for the semester are assigned under the principle of “first come, first served.”

For this study, only classes with a medium size (about 15–30 participants) and a weekly practice time of 60 to 90 min were selected, based on an agreement with the head of collegial sports (classes were a convenience sample, but participants were not self-selected). Class instructors were informed about the study and had to consent prior to asking individual participants of the course. Individual study participation was voluntary and the participant’s written consent was obtained prior to study begin. Nearly all class participants gave consent. However, due to data protection regulation, neither the complete list of class participants could be obtained and thus, nor the exact response rate. The study was approved by the Data Security Commissioner and the Ethics Committee of Bielefeld University.

The classes comprised various types of sports, such as aerobic exercise, including specific dance steps (Zumba, Bokwa), martial arts (Kickboxing, Taekwondo, Capoeira), Freeletics (a specific set of endurance and strength exercises), and basketball training. Course duration varied slightly. 13 weeks was the minimum duration at both universities, and only these weeks were considered for the statistical analysis, as only a few people attended more weeks.

Extensive prior knowledge about the expected effects at different levels as well as on variances and covariances is necessary to calculate the required sample size in mixed-effect models in order to attain sufficient statistical power. For our study, most of this knowledge was lacking. We aimed for a sample size of about 300 individuals from 10 gym classes. This was for practical reasons as well as to obtain a cluster size of about 30 at the second level and a sufficiently large sample size at the lowest level.

### Procedures

Individuals agreeing to participate signed a consent form when attending the class for the first week, and filled in a baseline questionnaire. All selected courses were attended on a weekly basis by student assistants who documented participation, issued a short questionnaire to all attending study participants when the session started, and collected them at the end of the session. Each participant had an individual, unique code consisting of letters and numbers of their family names, birth year, and place of birth in order to match the questionnaires to individual participants.

### Measures

Only measures relevant for the analyses depicted in this paper are described. The baseline questionnaire asked about the participant’s age (year of birth extracted from self-generated id code), gender, and student status (yes/no). To adjust for past behavior, participants were asked if they had already been exercising on a regular basis (yes/no) before registering for the class and if so, for how long (in months or years). Exercise was defined as any leisure time activities that included physical exercise, regardless of whether these activities were performed alone, in a team, or at a sports club. Some examples were given. For those who reported no previous regular exercise, months were set to 0.

Items for the weekly short questionnaire were based on existing studies and were adapted to the specific context of this study. Data on current affective states was collected at the beginning of the training, in about the midst of it (making use of short breaks for drinking or the like), and immediately after the end of the training, using two items, originally referring to the circumplex model of affect (Russell, 1980) which distinguishes between the two dimensions affective valence (pleasure vs. displeasure) and energetic arousal (low vs. high).

To explore whether the affective state immediately at the end of training has an impact on exercise class re-attendance, affect data collected at the end of the session was used for analysis (post-exercise affect). To incorporate changes in affective states in response to exercise we computed the difference in affect ratings as affective state at the end of a session minus state at the beginning of the session (Δ affect). Affective valence was measured using the Feeling Scale (Hardy and Rejeski, 1989), and for arousal, the Felt Arousal Scale was used (Svebak and Murgatroyd, 1985). The item for valence read “How do you feel at this moment?” and was answered on a scale from “very bad” to “very good.” In the original version, response options range from −5 to 5, but to fit with other scales, in our study it was adapted to the scale of 1 to 10. To measure arousal, we asked “How awake and active do you feel right now,” again with a 10-point-answer scale from 1 (“extremely tired”) to 10 (“extremely energized”). At the end of the weekly training, an additional item asked about the intention to re-attend the course the next week: “Do you intend to participate in this course again next week (next time)?” with possible answers ranging from 1 (“absolutely not”) to 10 (“at any rate”). This one-item intention measure was adapted from similar items of the theory of planned behavior (Bruijn et al., 2014).

Additionally, the weekly attendance of each participant was recorded by a student assistant on-site (1 = present, 0 = absent, or missing when class was canceled that week).

### Statistical analysis

For categorical data, descriptive statistics were calculated as percentages, and for continuous variables as means and standard deviations. In addition, the median is reported for very skewed variables. Attendance over time is described as the weekly participation rate, presented as the proportion of study participants attending a specific week of all participants who had the opportunity to attend. Some participants entered the study after the semester had already started, thus having fewer opportunities to participate. For each participant, the counting of weeks started with their first course attendance (“participant weeks” was defined as opportunities to attend an exercise class). All predictors were grand mean centered to predict weekly intention and re-attendance by affect. Additionally, for weekly measured variables, the variation was decomposed into a between- and a within-person-component. The between-component is the mean of the grand mean centered variable per person, while the within-component is the deviation of the weekly score from the overall mean per person (time-varying; Bolger and Laurenceau, 2013). Intention showed a highly skewed (“J-shaped”) distribution with mostly maximum intention values. Values were therefore transformed as 1/(11-intention)*10 before centering, arriving at a reduced skewness, using the same 1–10 range of the scale, and resulting in reasonably distributed residuals in the analyzed models.

All models included tests of time (participation week) and a quadratic time effect as additional predictors. There was also a 2-week holiday break at Christmas time during which classes did not take place. To consider the possibility of a change in intention or participation probabilities after this break, a further dichotomous variable, taking on the value “0” before Christmas break and “1” afterward, was included in the considered models (Singer and Willett, 2003; Bolger and Laurenceau, 2013).

To examine whether weekly affect values predicted the intention to re-attend at the next occasion, a hierarchical linear model was built in a stepwise manner. Participant week (level 1) was nested in ID (level 2) and class (level 3) and the models allowed for auto-correlated errors. The intra-class correlation coefficient (ICC) was calculated to show how much variance in intention can be explained by differences between participants (Bolger and Laurenceau, 2013). Restricted maximum likelihood estimation was used to estimate the model parameters. For model comparisons by likelihood ratio tests, the models additionally were fitted with ML. After fitting an empty (null) model as baseline for our comparisons, we first modeled the time-dependency of measures by comparing different constellations of time effects (see above). We then tested relevant background variables as potential confounders (university, gender, age, student status, and past exercise behavior before study start). When adding arousal variables to a model already including valence variables this caused multicollinearity problems. Since we laid our emphasis on valence, we restricted the models to this affect dimension. Post-exercise arousal and change in arousal during training were examined as supplementary model (see Supplementary material).

After adjustment of time course and background variables, post-exercise affective valence was entered as predictor (Model 1), and the change in valence during the class (Δ valence) was entered in a next step (Model 2). Random slopes were included for the within-components of the affect variables and for participation week. Additionally, the interaction effect between within-person and between-person components of valence was tested.

To predict participation in exercise classes throughout the term from weekly affect data, mixed-effect Cox proportional-hazard models were estimated as variant of survival analyses for recurrent events. The models predict the “hazard” to participate in the exercise class again (event) by considering the length of the time interval until this event occurs (time-to-event). Interval length was counted in opportunities to participate and constituted the time-to-event that represented the analyzed outcome. The resulting hazard ratio (HR) can be interpreted as the ratio of re-attendance rate at any given point in time that is associated with an increase in the corresponding predictor by 1, compared to the reference value. Therefore, a higher chance to re-attend can be seen as representing more regular class attendance.

In our sample, the event could happen every week, up to 13 times, but for most cases, at least some periods between events were longer. To the data, each person contributed up to 12 intervals between two attended classes, starting with the week of the first class attendance, so that the intervals were nested within the persons. Thus, we allowed for random effects of the person (level 2—also known as “frailty” in literature on survival models) and, additionally, the specific exercise class in which a person participated as another level of nesting (level 3). For example, a person attending a course every second week would contribute with several two-week intervals, a person attending only once at the beginning of the semester would contribute only a single open-ended interval, which is thus censored at the end of the course. Intervals were censored in case the information on the decision to re-attend the class was missing because the course (a) ended after about 3 months or (b) was intermittently canceled due to illness of the course instructor. In both cases, we had no information on whether a person would have possibly attended. Thus, information on if and when the event would have happened was missing. Every person was “at risk” of coming back after each attended (or intermittently canceled) class, i.e., a new time-to-event interval started. We used the Anderson-Gill approach to define the risk set for each interval (Andersen and Gill, 1982).

The proportional hazard assumption was tested by using standard survival models with an added frailty term to account for non-independence of observations of the same individual.

The statistical package R (R Core Team, 2018) was used for the calculations. Hierarchical linear models were estimated by the package “nlme” (Pinheiro et al., 2019), and, for mixed-effects Cox models, “coxme” (Therneau, 2019) was used for the survival analysis of recurrent events.

## Results

### Descriptive statistics

Of a total of 281 participants answering the questionnaires, 268 (4.6% excluded due to missing information) provided data on affective states and intention and were included in the analyses.

Nearly three quarters of the participants (*N* = 192, 68.3% of non-missing values) stated that they were already exercising on a regular basis. On average, they reported 91.2 months of regular exercise in the past (SD = 96.6, Median [Md] = 46, range = 0 to 384) and had 12.02 (SD = 2.79) opportunities to participate in the examined gym classes (range = 4–13), resulting in 5.17 (SD = 3.48) weeks of average class participation (Md = 4.5, range = 1–13).

Detailed descriptions of participation rate, intention, valence, and arousal over the 13 time points can be found in Table 1. The number of attending participants decreased strongly from 268 participants in the first week to 35 participants in the last week. The participation rate shows a relatively stable decline over time. Only slightly more than half of all participants re-attended the class as early as the following week. Overall, the participation pattern was irregular for many cases. Proportion of re-attendance increased slightly between participation week 8 and 11. For the majority of participants, Christmas holidays fell into this period. Therefore, the Christmas break was included in the survival analysis as a possible confounder, since it seems to predict the probability of participation.

**Table 1:** Participation in exercise classes and changes in psychological variables over time.

Week (interval)	*N*	*N* attended	Participation rate	*N* analyzed	Intention		Affect valence		Δ valence		Affect arousal		Δ arousal	
					M	SD	M	SD	M	SD	M	SD	M	SD
0	268	268	1.000	246	9.10	1.54	7.70	1.84	0.74	2.01	7.15	2.13	1.04	2.40
1	266	156	0.583	143	9.24	1.39	7.81	1.64	0.80	1.76	7.55	1.82	1.12	2.35
2	266	144	0.541	140	8.97	1.77	7.61	1.71	0.83	1.90	7.17	1.85	0.99	2.38
3	264	134	0.508	126	8.99	1.72	7.95	1.42	1.32	1.80	7.56	1.68	1.25	2.14
4	250	100	0.400	95	9.15	1.47	7.72	1.53	1.01	1.55	7.28	1.72	0.88	2.20
5	243	101	0.416	97	9.01	1.38	7.69	1.58	1.08	1.71	7.30	1.77	1.13	2.19
6	243	86	0.354	83	8.87	1.62	7.81	1.34	0.99	1.68	7.61	1.64	1.10	1.91
7	236	76	0.322	75	9.01	1.39	7.67	1.54	1.17	1.61	7.43	1.89	0.85	2.14
8	233	56	0.240	54	9.11	1.57	7.43	1.74	0.76	2.11	7.30	1.94	0.69	2.05
9	243	66	0.272	65	9.34	1.05	7.57	1.72	1.00	2.02	7.43	1.77	0.97	2.15
10	242	72	0.298	67	8.90	1.52	7.61	1.55	1.09	1.73	7.27	1.85	1.03	2.48
11	228	57	0.250	56	8.91	1.67	7.54	1.62	0.80	1.86	7.27	1.95	0.91	2.19
12	203	36	0.177	35	9.09	1.58	7.20	2.18	0.63	1.99	7.17	2.24	1.09	2.20

*N,* total cases still under observation; *N* attended, number of participants attending the class per occasion; *N* analyzed, number of cases that were analyzed in the prediction models after exclusion of cases with missing information on intention or affect variables; week, week of participation, starting with “0” for the week of first attendance of an individual and counting the weekly occasions to participate (censored cases are those where the course had ended); intention, raw score (untransformed), intention, valence and arousal values range from 1 to 10, M, mean, SD, standard deviation; Δ valence and Δ arousal, changes in affect from beginning to end of class.

Despite the irregular participation of most participants, intention to re-attend the class in the following week in general was rated very high with mean values around 9 (before transformation) on a scale from 1 to 10 for every week (overall mean = 9.06, SD = 1.54). No clear temporal pattern in intention was found when looking at the weekly mean values (see Table 1). However, these means only represent the intention of those participants who attended the class during the week in question and thus are not based on the same sample for each week. Therefore, the means cannot be interpreted as a within-person trajectory over time. Intention showed a small peak around the same time as participation rate. So, the Christmas break was also factored in when modeling temporal changes in intention.

Affective valence at the end of the training was also rated relatively high, with means near 8 again on a 1-10-point scale. There was a slight decrease in valence over the term (from M = 7.70 [SD = 1.84] at week 0 to M = 7.20 [SD = 2.18] at week 12). Arousal followed a parallel pattern for most weeks, with slightly lower values than valence, but no clear temporal trend. In terms of the changes in affect during the training, valence as well as arousal on average showed increases of about one point from pre- to post-exercise. Again, no clear temporal trends were revealed over the semester.

### Prediction of intentions to attend the class again

#### Model building

The 1,282 observations were nested in 268 individuals that were in turn nested in 10 exercise classes. Therefore, a 3-level model was examined first. However, since the between-class variation in intention was comparably negligible (about 0.7% of explained variance) and neither AIC nor BIC showed an improvement, we stuck to the 2-level-models. For intention, ICC was 0.462 without auto-correlation, and 0.395 when allowing for auto-correlated errors (Phi = 0.348). A model with auto-correlated errors of first order (form = ~week|person) fitted the data better than without (likelihood ratio test: χ^2^_(df = 1)_ = 49.99, *p* < 0.001). Thus, after accounting for the correlation structure, about 39.5% of the total variance was between persons.

To adjust for the time course of intentions, a model including participation week and a binary predictor distinguishing between the period before and after Christmas holidays showed the best fit. University, gender, age, and past exercise behavior were not significant confounders (results not shown). Therefore, they were excluded from the models to avoid overly complex models and accumulating missing values. Random slopes for the weekly measured variables improved the model (model comparison against random intercept model: χ^2^_(df = 9)_ = 81.09, *p* < 0.001), thus random slopes were included.

#### Model results

Table 2 shows the effects of the within- and between-predictors related to affective valence on weekly intention. In model 1, post-exercise valence was the only predictor after having adjusted for time effects. Higher affective valence at the end of the training was significantly associated with a higher intention to re-attend the next week. Results were significant on both within- (level 1) and between-subject (level 2) levels. In detail, in those weeks in which valence was higher than in the others, the intention was also higher within a specific person (“valence within”). Practically speaking, a one-point increase on the valence scale (range 1 to 10) was associated with a 0.721 increase on the intention scale (range 1–10; *p* < 0.001). There was also a significant association with the average valence over the course of all weeks (“valence between”): Those participants with a higher mean valence also reported higher weekly intentions (a one-point increase in valence was related to an estimated increase of 0.793 on the intention scale). There was no interaction effect between within and between components of valence revealed.

**Table 2:** Results of hierarchical linear models for the prediction of weekly intention by affect.

	Model 1 (post-exercise valence)	Model 2 (post-exercise valence + Δ valence)
**Fixed effects: *β* (95% CI)**		
Intercept	7.112 (7.451–7.790)	7.438 (7.098–7.778)
Valence between	0.793 (0.584–1.001)^***^	0.880 (0.647–1.114)^***^
Valence within	0.721 (0.553–0.889)^***^	0.831 (0.645–1.017)^***^
Δ valence between	/	−0.217 (−0.460–0.026)^#^
Δ valence within	/	−0.165 (−0.271–0.059)^**^
**Random effects: variance (95% CI)**		
Valence within	0.543 (0.326–0.905)	0.604 (0.330–1.106)
Δ valence within	/	0.013 (0.001–0.185)
Residual variance level 2 (person)	4.281 (3.061–5.986)	4.425 (3.185–6.147)
Residual variance level 1 (time)	4.626 (3.983–5.373)	4.510 (3.894–5.222)
AIC	6025.205	6023.546
BIC	6092.236	6121.513
LL	−2999.603	−2992.773

One thousand and two hundred and eighty two (1,282) observations nested in 268 individuals; LL, log-likelihood; AIC, Akaike Information Criterion; BIC, Bayes Information Criterion; Δ, difference in valence as valence post-exercise minus valence pre-exercise. All results are controlled for time course (participation week and time period before/after Christmas).

# *p* ≤ 0.10;

** *p* ≤ 0.01;

*** *p* ≤ 0.001.

In model 2, change in valence was entered as a second predictor. Overall, the model fit improved slightly in terms of the likelihood and AIC, while BIC clearly preferred the simpler model without the change in valence. A higher increase in valence was associated with lower subsequent intention, although the between-person effect was only marginally significant (*p* < 0.10). Individuals with a one unit larger average increase on the valence scale were expected to show an intention reduced by 0.22 (*p* < 0.10), and within-person an increase one point higher than in the average week resulted in an expected reduction of 0.17 for intention to re-attend (*p* < 0.05).

We found an overall correlation between post-exercise valence and Δ valence of r = 0.52. This means that, in general, a negative affective response (Δ) resulted in a lower valence post-exercise or, to put it the other way round, a more positive response was accompanied by a more positive affective state post-exercise. Both variables therefore shared some information but also provided enough independent contributions to be able to distinguish the effects.

The supplementary models showed a significant increase in intention also with higher post-exercise arousal between and within. In contrast, a higher increase in arousal during class time, similar to valence, resulted in a somewhat lower intention to re-attend (see Table A in Supplementary material). Random slopes indicated that both valence and arousal effects varied between persons.

### Prediction of weekly re-attendance

#### Model building

To predict participation in the exercise class from affect, affective states at the end of each attended training were used as predictors of the hazard to re-attend the class in a mixed-effect Cox proportional hazard model. To use the same variable as predictor that had been the outcome of the linear models described above (see Table 2), weekly intention was not decomposed into between- and within-variance components for the main analysis.

For participation, the temporal course was best described by including week and a quadratic term of week as predictors in the Cox model, meanwhile the Christmas holidays were not meaningful in this case. Unlike in the linear models, including a random effect for class improved the models, so it was included as a third level. Gender was the only potential confounder significantly associated with the outcome (with the chance of re-attendance nearly 20% lower in females than in males). Since including gender violated the Cox proportional hazard assumption, gender was included as a stratification variable in the final models instead of a potential confounder. This results in allowing different baseline hazards for males and females while other results, i.e., the tested effects, remained unchanged. Due to the complexity of the models, no random slopes could be included in the final models. However, we additionally separately analyzed random slopes, varying by course or person, for the affective variables to get an impression of the variability of effects.

**Table 3:** Results of Cox multilevel survival models for the prediction of re-attendance by affective valence.

	Model 1 (post-exercise valence)	Model 2 (+ Δ valence)	Model 3 (+ intention)	Model 4 (intention decomposed + interactions valence × intention)	Model 5 (parsimonious model)
**Fixed effects of predictors: HR (95% CI)**					
Valence between	1.040 (0.974–1.111)	1.031 (0.960–1.108)	0.989 (0.919–1.065)	0.980 (0.907–1.059)	0.997 (0.931–1.068)
Valence within	1.059 (0.998–1.124)^#^	1.084 (1.008–1.167)^*^	1.055 (0.980–1.136)	1.053 (0.978–1.135)	1.036 (0.976–1.100)
Δ valence between		1.020 (0.948–1.097)	1.030 (0.959–1.106)	1.039 (0.967–1.117)	/
Δ valence within		0.969 (0.914–1.028)	0.977 (0.922–1.036)	0.978 (0.922–1.036)	/
Intention (weekly)			1.052 (1.028–1.077)^***^	*within:* 1.051 (1.019–1.084)^**^ *between:* 1.048 (1.012–1.085)^**^	*within:* 1.051 (1.019–1.084)^**^ *between:* 1.046 (1.010–1.083)^*^
Intention × valence between				0.978 (0.958–0.999)^*^	0.979 (0.959–1.000)^*^
Intention × valence within				1.009 (0.986–1.032)	/
**Random effects (variance intercept)**					
Individual	0.157	0.154	0.144	0.143	0.146
Class	0.081	0.081	0.078	0.071	0.069
**Model fit**					
AIC	8512.062	8514.645	8498.069	8498.770	8495.086
BIC	8541.604	8554.034	8542.382	8557.854	8539.399
Integrated LL	−4250.031	−4249.322	−4240.035	−4237.385	−4238.543

N events, 1,016, N intervals, 1,282 entered as “start” and “stop” week; HR, hazard ratio; LL, log-likelihood; AIC, Akaike Information Criterion; BIC, Bayes Information Criterion. All models were adjusted for time course (participation week and participation week squared) and stratified by gender.

# *p* ≤ 0.10;

* *p* ≤ 0.05;

** *p* ≤ 0.01;

*** *p* ≤ 0.001.

#### Model results

In the first model (model 1; Table 3), post-exercise affective valence was entered as a predictor. A significant effect was only found for within-person fluctuations of valence from week to week, while the average level of valence of a person over the term (“valence between”) had no meaningful effect. That is, adjusted for the temporal pattern in participation and stratified for gender, persons with a higher average valence level were not more likely to re-attend, but participants were slightly more likely to re-attend after weeks in which they rated valence higher than in other weeks although the effect was only marginally significant (HR = 1.059, *p* < 0.10).

In model 2, the change in valence from beginning to end of training was added to the model as further affect variable. Only within-person fluctuations of post-exercise valence predicted subsequent re-attendance, while between-person differences and both components of change in valence turned out to be clearly not significant (*p* > 0.10). Specifically, for each one-point increase on the post-exercise valence scale, the chance (“hazard”) to re-attend the class increased by 8.4% (HR = 1.084, *p* < 0.05). However, after adjusting for intention in model 3, the effect of valence within decreased in magnitude, and none of the valence variables showed a statistically significant HR. No effect of arousal was detectable in a model excluding valence (see Table B in Supplementary material). Hypothesis 3, which stated a direct positive effect of the affective state or response on re-attendance, was therefore only partially supported for the within-person component of affective valence.

In contrast, higher weekly intention was associated with a significantly higher probability of re-attendance: A one-point increase on the (transformed) intention scale led to a 5.2% increased hazard for re-attendance. The fact that the inclusion of intention resulted in non-significant valence effects points to an indirect effect of valence where intention partially mediates the effects of within-person fluctuations in affective states: the hazard ratio for valence decreased from 1.084 to 1.055 when intention was entered.

In model 4, intention was also decomposed, and the interactions between weekly intention and affect (within and between-persons) were added. For intentions, both within- and between-person component effects were very similar to the overall variable effect. None of the valence variables was significant after this adjustment but the interaction term of intention with valence between-persons was. It showed that the effect of intentions was getting smaller with a higher valence. For example, with a mean valence value higher than that of 90% of the sample the HR of intention was only 1.008, while for an individual belonging to the 10% of those with the lowest valence the predicted HR could reach 1.081, which means an increase in the hazard rate of about 8% when intentions are increased by 1 point. Thus, hypothesis 2, which proposed a moderator effect of affective state, was not supported since it states a *larger* effect of intention with more positive affective response. Only small interaction effects with intention in different directions were revealed for arousal (see Table B in Supplementary material).

In an additional model (model 5), we only kept the post-exercise valence and excluded other non-significant variables not part of the interaction to arrive at a more parsimonious model. This model did equally well as the full model (Likelihood ratio test: χ^2^_(df = 3)_ = 2.32, *p* = 0.51) and was the best in terms of both information criteria.

Analyses on random slopes showed that the effect of affective valence within persons mainly varied between the courses (from HR = 0.86 to 1.19), but not persons.

## Discussion

The purpose of this study was to examine the role of affective states for exercise intentions and class re-attendance. Overall, though affective states were related to both, not all hypotheses were backed by the data. The following discussion will be structured around the three tested hypotheses.

### Hypothesis 1: Affect and exercise intention

The first hypothesis stated that affective states predict intentions to re-attend the exercise class at the next occasion. Hypothesis 1 was confirmed in terms of affective valence on the within- and between-person level.

The effect on the between-level suggests that people who on average feel better than others after exercising have higher intentions to exercise again. The effect on the within-level supports the notion that exercise units with more positive valence lead to stronger immediate intentions to re-attend the exercise class in the following week. A larger increase in valence during the class, when added to the model, however, was associated with a slightly lowered intention, especially within-persons. Other than expected, this effect shows that in those weeks when individual affective valence showed a larger increase than usual and, therefore, a more positive affective response, this coincided with lower motivation to re-attend, at least after adjusting for absolute valence post-exercise. In fact, in a model without post-exercise valence, a positive change in valence was associated with higher intention, albeit less pronounced than for post-exercise valence. Since in general a larger increase correlated with a higher post-exercise valence, the negative difference effect at constant post-exercise valence also points to lower valence at the beginning being associated with smaller intention at the end.

Our results are in contrast to review results, which conclude that, overall, during or post-exercise affective responses in terms of valence do not show meaningful effects on subsequent intentions (Rhodes and Kates, 2015), although in some individual studies the expected associations were found. For example, a study using similar measures as we did showed, in a sample of socially physique anxious female college students, that affective valence and arousal were significantly related to future intentions with valence being the more powerful predictor (Raedeke et al., 2007). This was the only other study we found which also asked for the intention to re-attend an exercise class specifically. Most other studies focused on more general intentions on future PA.

In a sample with a similar age as in our study, changes in positive or negative affect during exercise were indirectly related to intention, mediated by other TPB-constructs (not measured in our study), and for baseline valence an additional direct effect was revealed (Kwan and Bryan, 2010a). However, affect was used to predict exercise intentions 3 months later and not for the next occasion (which is usually within the next 1 or 2 weeks for regular exercise classes). The results are therefore not directly comparable.

In general, we are not aware of other studies showing negative effects of increasing valence. But, as mentioned above, we found this negative effect of increasing valence only for constant post-exercise valence (i.e., adjusted for post-exercise valence) where it also implies a lower valence at the beginning of the exercise session. This might be seen as confirming the role of baseline valence in the study of Kwan and Bryan (2010a). However, the studies differ in various aspects.

Overall, the confirmation of the hypothesis that more positive affective states result in higher intentions to re-attend the class at the next occasion in our study is in line with feedback theory of Baumeister et al. (2007) and dual process approaches like the PAAM model (Strobach et al., 2020). These state that automatic (implicit) affective valuations besides an implicit pathway of behavior regulation can be further processed by the reflective system and result in conscious motivation (like intentions).

In our study, arousal as additional affect dimension, when unadjusted for valence also showed significant associations with intentions at the within- as well as between-person-level (see Supplementary material), which was unexpected because of the assumed neutral valence. It was, however, highly correlated with valence and effects, therefore, may mirror those of a positive valence.

### Hypothesis 2: Affective states as moderators of the intention-behavior relationship

The second hypothesis stated that affective states moderate the relationship between intention to exercise and the actual re-attendance of the class in that more positive affect increases the chance that intentions are implemented in actual behavior. This hypothesis is part of the PAAM model (Strobach et al., 2020). No significant moderator effects could be found in our study, neither at the within- nor at the between-person level. The found interaction effect for valence and intention within, on the contrary, pointed to a slightly lower chance of high intentions leading to behavior with a more positive valence, while intention was still an independent significant predictor of behavior. The effect of short-term exercise intentions on behavior, therefore, might not depend on post-exercise affective states or changes in affect due to exercise. From our study, it can be concluded that affective valence supports the building of intentions, but do not facilitate the translation of intentions into behavior. The supplementary results for affective arousal were similar, although an additional small interaction for change in arousal was found.

Only one other study on the moderating effect of affect was found, which, in contrast to our study, confirmed the expected interaction between positive affect and translation of intentions into behavior (Kwan and Bryan, 2010b). However, its methods and design are not comparable to those of our study. Affective states were measured within different affect categories under lab conditions. Exercise behavior was measured 3 months after the measurement of affect and intention and operationalized as voluntary exercise frequency within the last 3 months. Therefore, only between-subject effects, but not within-subject effects, could be estimated, while our main focus was on the latter.

An explanation to why the results were not significant in the current study could also lie in the fact that exercise was objectively measured through observing re-attendance of the class and not by self-report questionnaires. We suppose that other results might support the moderation hypothesis, at least partially, due to a method effect. Measuring several constructs with the same method (e.g., questionnaire) might induce common variance between constructs, which can be caused by inter-individual differences in the response tendencies (Bolger and Laurenceau, 2013). Nevertheless, further studies are required to clarify this issue.

Getting back to the PAAM model, this stresses habits as further important implicit concept besides affect. It predicts that positive affective response would increase habit strength (i.e., automaticity of behavior) and this increases the chance of enacting exercise or other PA behavior. Habit was not included in the presented analyses. But we found support for affective valence post-exercise as well as an increase in valence during exercise promoting the development of automaticity of the decision to attend an exercise class in a previous analysis (Weyland et al., 2020). The possible indirect effect of affective valence on behavior *via* habit strength postulated by the PAAM should be tested in future studies.

### Hypothesis 3: Direct effects of affective states on subsequent behavior

The third hypothesis stated that affective states directly impact on the probability of actual re-attendance of the class at the next occasion, thus referring to a more regular class attendance. This would be in line with hedonic and some dual process theories, namely that affective valence is directly related to future exercise behavior *via* an implicit, associative learning pathway (Williams and Evans, 2014; Brand and Ekkekakis, 2018; Stevens et al., 2020).

Our data supported this hypothesis only partially. First, although intra-individual fluctuations in post-exercise valence were related to re-attending the class in the first survival models we tested, no meaningful effect was revealed on the between-person level or for changes in valence during the class. Second, even this effect could not be demonstrated after adjusting the valence effects for intentions in the more comprehensive models.

This means that immediate post-exercise valence predicted exercise class re-attendance independent of the baseline valence level. And this effect did not depend on the average level of valence, but related to short-term intra-individual fluctuations in affective experiences relative to this average level of a person: after those exercise units, in which affective valence was more positive, the likelihood for re-attendance increased. Therefore, situational factors concerning affective states in single exercise units may be more important for exercise class re-attendance than stable inter-individual differences. The fact that supplementary analyses mainly showed variations in the valence effect between different courses indicates that, besides occurring differences in participation rates between courses, characteristics of the type of exercise or trainer, which were not analyzed in our study, may have impacted the affect-behavior relationship.

Other studies support direct effects of affective responses during exercise but not post-exercise on future behavior (for an overview see Rhodes and Kates, 2015; Stevens et al., 2020). However, the analyzed effects were in general not adjusted for intentions.

In a study by Kwan and Bryan (2010b) increases in positive affect during exercise were related to a higher PA frequency at 3 months follow-up. No such association was found for affective responses (positive or negative affect) 15 min post-exercise. Williams et al. (2012) showed that valence during and immediately after a 10-min treadmill walk predicted subsequent self-reported lifestyle PA up to 6 months later. The predictive potential of valence for PA behavior was also confirmed in other studies (e.g., Williams et al., 2016), while further studies confirmed the relationship for implicit (affective) attitudes as related concept with PA (Conroy et al., 2010; Hyde et al., 2012).

Overall, a direct effect of affective response during exercise on future PA behavior can be assumed from previous research (without adjustment for intentions). For post-exercise affect there seems to be a difference between measuring valence immediately after exercise versus after a time delay after ending of activity. As for example Williams et al. (2012) did, we measured valence immediately at the end of training. It is theoretically assumed that to measure affective state itself it has to be *in vivo* during an activity because a retrospective evaluation would be further cognitively processed and relate to the reflective path in terms of dual process models (Williams and Evans, 2014; Brand and Ekkekakis, 2018; Stevens et al., 2020). We think that a measurement immediately after the activity still captures the acute affective state and the difference from beginning of a class to this point relates to the acute affective response. Moreover, according to the peak-end-rule in hedonic theory (Kahneman et al., 1993, 1999) affective experiences at the end of a behavior, besides those with the highest intensity, are critical for overall evaluations and future behavior.

To complete a questionnaire while exercising was not possible in the kind of group activities we observed without interrupting the class. So, even measurement earlier in class time would have taken place while taking a break from activity. We cannot rule out the possibility that knowing that the training has been accomplished was a factor that also impacted on the affect measurement (rebound effect). However, in general, valence in the midst of the class (during a break) was very similar to our post-exercise measurement. This was also mentioned by Kwan and Bryan (2010a). On average, during class valence increased in our sample and, therefore, did not show the characteristic decline which would be expected at high-intense exercising (Williams, 2008).

Though a difference between in-task and post-task affect seems obvious from existing studies, the time points for the post-measurement differed between studies (Rhodes and Kates, 2015), and the exact point in time when a retrospective evaluation as opposed to an acute affective response is to be assumed has still to be determined.

The non-significance of valence variables in our study after adjusting for intentions points to an indirect rather than a direct effect of valence on re-attendance. It seems as if the increase in intention explains a large portion of the found effect on re-attendance. When looking at Figure 1, this indirect path mediated by intention is implied by hypothesis 1, which was confirmed, while the (additional) direct path proposed by hypothesis 3 was not.

Besides possible indirect effects *via* habit (implicit path) as described above, indirect effects of affective valence *via* intentions (reflective path) should be further explored in the future.

We analyzed affective arousal separately in supplementary models because of its high correlation with valence. We did not find any direct association of arousal variables with re-attendance, neither for the effects unadjusted, nor adjusted for intention.

### Strengths and limitations

A strength of this study is the continuous weekly measurement of exercise class attendance over a period of one semester. In this manner, exercise behavior could be observed over the course of several months. The effects of affective states could be differentiated on the between-subject and within-subject level, providing valuable knowledge on affective and motivational processes. Furthermore, PA attendance was measured quasi-objectively by observation. In this way, systematic bias of subjective measures of PA could be avoided (Jekauc et al., 2014). However, there are also some limitations to consider.

A limitation of this study concerns the high percentage of missing data, which is mainly due to low participation and high dropout rates (Jekauc et al., 2012). We therefore adapted our analysis and used a survival model with time-to-event intervals of different lengths, instead of predicting weekly re-attendance. However, we have no data on participants dropping out, nor on the reasons for their dropout. It cannot be assumed that not attending the exercise class is equivalent to not adhering to exercise, as there is no data on whether a person was exercising in another class or was active otherwise in his or her leisure time. However, the intention we asked for and the behavior we were interested in were directly related to re-attending this specific exercise class, but not to the overall amount of PA. We therefore precisely observed the behavior that the intention item referred to.

Another limitation is that intention to re-attend was not predicted over time, but measured at the same instant as affective states post-exercise. The association with affective states may therefore be overestimated, especially as feeling states might overall impact response behavior. Furthermore, no causal inferences are possible. It is likely that affect not only influences intentions, but that a reciprocal effect exists, which previously was not considered (Kwan and Bryan, 2010a). Intentions as well as affective states were measured on single-item scales with uncertain measurement properties, since only very short weekly questionnaires were possible for the benefit of repeated measurements. Although the scales used for affect variables are well established internationally, no validated German version existed at the time of data collection. Moreover, we adjusted the answer scales of the Feeling Scale and the Felt Arousal Scale to parallel those of other items (10-point scale) while the original scales are used with a 6 or 11-point scale. This was done to allow for an easy and fast completion of the short questionnaires during class time. Evmenenko and Teixeira (2020) additionally point out that the usefulness of these single-item affect scales might vary, depending on the method of application and kind of activity. Although not common practice, exercisers may also profit from a training in understanding and answering the scales (Ekkekakis, 2013), and no standard in terms of the exact best time points for measurement during or post-training exists, since these may also depend on the activity itself.

Especially the intention item was very skewed, indicating a ceiling effect. It could be used after a transformation, resulting in approximately normally distributed model residuals. However, the development of reliable and valid short scales is indicated for future studies with frequent measurements. A recent study by Maibach and colleagues validated a German version of both affect scales by analogy with similar studies on the original English versions. It found good convergent validity, comparable to the original versions, using a non-verbal criterion (Maibach et al., 2020). Since the Felt Arousal Scale was validated with a 6-point answer scale and the bipolar scale was translated differently from ours, we cannot be sure to have used a valid version that reflects the same construct as the validated German or the original English version. Unfortunately, the validation took place after we had already finished our data collection. For future German studies, employing these validated versions of both scales would be recommended.

Due to high correlations between the time points within an exercise class, we could not include different time points of affect measurement into one model or adjust for baseline exercise affect, for example. Previous research found affective states during exercise more relevant than post-exercise affective states. We included affect measurement immediately after training and the change from pre- to post-exercise as measure for affective response to exercise. This point was discussed above in the context of hypothesis 3. The inclusion and comparison of different measurement points would be preferable in future studies to better represent affective responses to exercise. However, we repeated our analyses employing valence measured midst-session instead of post-exercise, and the results were very similar with the exception that within-person effects were smaller (results not shown).

Last but not least, since our sample mainly consisted of university students, the generalizability of the results to other population groups is questionable. However, besides the fact that no meaningful effects of student status were found in our analysis of potential confounders, most theories explaining PA intentions and behavior were developed to apply to all people but not only to specific subpopulations. As such, theoretical hypotheses derived from such theories should hold for different population groups, including university students. Furthermore, university students are generally young and well-educated, and both characteristics are deemed to make regular exercising more likely. Yet, our study points to a large intention-behavior-gap even in this population and this despite high intention values. The questions addressed thus seem very relevant for this group, too. Nevertheless, although valence and especially intention showed statistical significant effects, they cannot explain the high attrition in our study.

### Implications

Notwithstanding these limitations, some future research and practical implications can be derived from this study. Exercise promotion interventions and instructors should structure exercise programs in order to attain a positive affective response especially post-exercise, considering the effects of affective states on intentions and behavior. Affective responses to exercise seem to vary with exercise intensity. In general, intensities below the ventilatory threshold elicit positive affective responses, and self-selected exercise intensity seems preferable for valence (Williams, 2008).

While it is still difficult to plan an exercise program that elicits positive affective responses in each individual, some studies found positive results for changing social and environmental factors (Jekauc, 2015). An experimental study provided evidence that a systematic manipulation of teacher’s feedback led to a change of perceived competence, which in turn supported the development of positive or negative affective states in physical education (Bruijn et al., 2014). Hence, teachers and coaches may play a crucial role in the promotion of positive affective states. Promotion of health, rather than an appearance oriented leadership style, led to a better affective response (Raedeke et al., 2007), as well as exercising in a group of people compared to exercising alone in other research (Dunton et al., 2015; Graupensperger et al., 2019). Another promising approach would be to educate teachers (Leisterer and Jekauc, 2019) and coaches (Hagger et al., 2002; Strauch et al., 2018) in social–emotional skills, which in turn have been shown to be related to positive affective states of participants (Strauch et al., 2018). Regarding research, future studies should investigate the relationship between affective states, intention, and exercise adherence by continuous measurements over a longer period of time (e.g., 6 months or longer). This would allow for an examination of the effects of affective states on long-term maintenance, which have not been examined yet. Furthermore, studies should include measures for non-participating individuals and dropouts in order to discover the underlying mechanisms. This could be realized, for example, by ecological momentary assessment or online-questionnaires although, presumably, regular answers would have to be encouraged by incentives to achieve the drop outs.

## Conclusion

The results of this study suggest that more positive affective state, especially immediately after training, supports the process of the formation of short-term exercise intentions which, in turn, are predictive of subsequent exercise class attendance. However, we could not find evidence that affective states moderate the intention-behavior relationship in that a higher valence relates to more emphasized intention effects and therefore helps closing the intention-behavior-gap. Rather, affect-behavior relations seemed to be mediated by intention. Although this does not conform to the tested hypothesis, the possibility of a mediated effect is also implied by dual process approaches. Further studies should further explore the possible indirect effects of exercise-induced affects *via* the implicit versus explicit pathway, which are postulated by dual process theories.

As a consequence, interventions should be developed, that promote positive affective responses, and education curricula should be extended to promote social–emotional competences in coaches and physical education teachers. However, further longitudinal studies with frequent measurements are needed.

## Data availability statement

The raw data supporting the conclusions of this article will be made available by the authors upon request, without undue reservation.

## Ethics statement

The studies involving human participants were reviewed and approved by Ethics Committee of Bielefeld University (Bielefeld, Germany). The participants provided their written informed consent to participate in this study.

## Author contributions

EF designed and coordinated the study, supervised data collection, conducted the statistical analysis, and wrote the paper. CN, SW, and BW wrote the paper. OS helped with the analysis. DJ designed and coordinated the study and wrote the paper. All authors contributed to the article and approved the submitted version.

## Funding

We acknowledge support for the Article Processing Charge by the Deutsche Forschungsgemeinschaft and the Open Access Publication Fund of Bielefeld University.

## Conflict of interest

The authors declare that the research was conducted in the absence of any commercial or financial relationships that could be construed as a potential conflict of interest.

## Publisher’s note

All claims expressed in this article are solely those of the authors and do not necessarily represent those of their affiliated organizations, or those of the publisher, the editors and the reviewers. Any product that may be evaluated in this article, or claim that may be made by its manufacturer, is not guaranteed or endorsed by the publisher.

## Abbreviations

PA: Physical activity
HR: Hazard ratio
