# Supplementary material for: Examining the role of affective states in relation to exercise intentions and participation in extra-curricular exercise classes at university: A repeated measurement observational study
Source: Front Psychol. 2022 Aug 22;13:815466. doi: 10.3389/fpsyg.2022.815466 (PMC9443811; doi:10.3389/fpsyg.2022.815466)
Supplement: Supplementary file 1 [file Data_Sheet_1.PDF]

Supplementary Material

Table A: Results of hierarchical linear models for the prediction of weekly intention by affective arousal

	**Model 1** (arousal end of exercise class)	**Model 2** (post-exercise arousal + affective response (Δ arousal)
**Fixed effects:** (95% CI)		
Intercept	7.509 (7.154 - 7.864)	7.538 (7.186- 7.890)
arousal between	0.567 (0.373- 0.761)***	0.729 (0.492- 0.966)***
arousal within	0.458 (0.325- 0.591)***	0.462 (0.310- 0.613)***
Δ arousal between	/	-0.268 (-0.492- -0.045)*
Δ arousal within	/	-0.088 (-0.178- 0.003)#
**Random effects:** variance (95% CI)		
Arousal within	0.249 (0.130- 0.476)	0.238 (0.122- 0.466)
Residual variance level 2 (person)	4.904 (3.537- 6.800)	4.775 (3.457- 6.596)
Residual variance level 1 (time)	4.896 (4.245- 5.647)	4.861 (4.225- 5.594)
**Model fit:**		
AIC	6090.951	6071.954
BIC	6157.961	6149.249
LL	-3032.476	-3020.977

Note. 1,282 observations nested in 268 individuals; # ≤ .10, * p ≤ .05, ** p ≤ .01, *** p ≤ .001; LL: log-likelihood; AIC: Akaike Information Criterion; BIC: Bayes Information Criterion; Δ: difference in arousal as arousal post-exercise minus arousal pre-exercise.

All results are controlled for time course (participation week and time period before/after Christmas).

Table B: Results of Cox multilevel survival models for the prediction of class re-attendance by affective arousal

	**Model 1** (post-exercise arousal)	**Model 2** (+ Δ valence)	**Model 3** (+ intention)	**Model 4** (intention decomposed + interactions arousal × intention)[a]
**Fixed effects of predictors: HR (95% CI)**				
Arousal between	1.039 (0.981-1.100)	1.054 (0.984-1.130)	1.020 (0.950-1.094)	1.027 (0.955-1.104)
Arousal within	0.991 (0.940-1.045)	1.007 (0.943-1.076)	0.985 (0.923-1.052)	0.985 (0.923-1.052)
Δ arousal between		0.976 (0.914-1.042)	0.990 (0.928-1.056)	0.977 (0.914-1.044)
Δ arousal within		0.983 (0.936-1.032)	0.986 (0.939-1.035)	0.986 (0.939-1.035)
Intention (weekly)			1.055 (1.031-1.080)***	*within:* 1.059 (1.027-1.093)*** *between:* 1.045 (1.009-1.082)*
Intention × arousal between				0.974 (0.951-0.998)*
Intention × Δ arousal between				1.027 (1.001-1.053)*
**Random effects (variance intercept):**				
individual	0.154	0.158	0.149	0.144
class	0.081	0.079	0.081	0.074
**Model fit**				
AIC	8493.881	8478.140	8458.467	8497.415
BIC	8523.411	8517.497	8502.745	8517.548
Integrated LL	-4240.941	-4231.070	-4220.234	-4217.256

Note. N events = 1016, N intervals = 1282 entered as 'start' and 'stop' week; # p ≤ .10, * p ≤ .05, ** p ≤ .01, *** p ≤ .001; HR = hazard ratio; LL = log-likelihood; AIC = Akaike Information Criterion; BIC = Bayes Information Criterion; [a]: interactions of intention with arousal or Δ arousal within were not significant.

All models were adjusted for time course (participation week and participation week squared) and stratified by gender. According to Information criteria, the more parsimonious model 3 without interaction terms was favored.
